# SP1 Expression and the Clinicopathological Features of Tumors: A Meta-Analysis and Bioinformatics Analysis

**DOI:** 10.3389/pore.2021.581998

**Published:** 2021-01-28

**Authors:** Yue Gao, Kai Gan, Kuangzheng Liu, Bin Xu, Ming Chen

**Affiliations:** ^1^ Surgical Research Center, Institute of Urology, Medical School of Southeast University Nanjing, Jiangsu, China; ^2^ Department of Urology, Affiliated Zhongda Hospital of Southeast University, Nanjing, Jiangsu, China

**Keywords:** specificity protein 1, cancer, clinicopathological characteristics, prognosis, biomarker

## Abstract

**Objective:** Specificity protein 1 (SP1) plays a vital role to promote carcinogenesis in a variety of tumors, and its up-regulated expression is reported to be a hinter of poor prognosis of patients. We conducted this meta-analysis to elucidate the clinical significance and prognostic value of SP1 in malignant tumors.

**Methods:** PubMed and Cochrane Library were searched for studies published between January 1, 2000 and June 1, 2020. The combined odds ratios (ORs) and hazard ratios (HRs) with 95% confidence intervals (95% CIs) were used to investigate the correlation of SP1 with clinical behaviors and prognosis in patients with solid tumors. UALCAN was used to conduct bioinformatics analysis.

**Results:** A total of 24 documents involving 2,739 patients were enrolled in our review. The random-effect model was used to perform this analysis due to the high level of heterogeneity. SP1 low expression was not conducive to lymph node metastasis (OR = 0.42; 95% CI: 0.28-0.64; *p* < 0.05), progression of TNM stage (OR = 0.34; 95% CI: 0.20-0.57; *p* < 0.05) and tumor infiltration (OR = 0.33; 95% CI: 0.18-0.60; *p* < 0.05). Elevated SP1 expression was connected with shorter survival time of patients with hepatocellular carcinoma, pancreatic cancer, gastric cancer and esophageal cancer (HR = 1.95; 95% CI: 1.16-3.28; *p* < 0.05). According to UALCAN database, breast cancer, ovarian cancer, colon cancer and lung adenocarcinoma display an elevated SP1 expression in comparison with normal tissues. Kaplan-Meier survival plots indicate SP1 mRNA level has negative effects on prognosis of liver hepatocellular carcinoma and brain lower grade glioma.

**Conclusion:** SP1 was associated with lymph node metastasis, TNM stage and depth of invasion, and indicated poor clinical outcome, which brought new insights on the potential candidacy of SP1 in clinical usage.

## Introduction

Specificity protein 1 (SP1) serves as a transcription factor involved in transcription of a large amount of “housekeeping genes”, regulating cell proliferation, differentiation and apoptosis [1]. It promotes gene transcription by directly binding to genes in GC-rich elements of promoters three C2H2-type zinc fingers. Many housekeeping genes play central roles in tumor formation and progression. As a consequence, SP1 is closely related to aggressive tumor behaviors and poor outcomes.

SP1 exerts its biological function in many ways like epithelial-mesenchymal transformation (EMT), angiogenesis, inflammatory signaling and immune escape. It has been confirmed that SP1 can regulate a variety of cancer-related genes. SP1 controls the proliferation of breast cancer cells by interacting with insulin-like growth factors I receptor (IGFIR) [2]. Besides, SP1 binds to the promoter of vascular endothelial growth factor (VEGF) to facilitate angiogenesis, forming a favorable condition for tumor growth [3]. In lung cancer, SP1 participates in the induction of matrix metalloproteinase-2 (MMP-2) and matrix metalloproteinase-9 (MMP-9) to accelerate cell invasion [4].

Many tumors show increased SP1 expression, including breast cancer, gastric cancer, lung cancer and pancreatic cancer, compared with adjacent healthy tissues. In cervical cancer, overexpression of SP1 activates POU3F3 to promote the proliferation and invasion [5]. SP1 can also interact with Ajuba to form a complex to induce downstream gene transcription, contributing to an unsatisfactory outcome to pancreatic cancer patients [6]. SP1 level affects tumor stage and differentiation, which implies an unfavorable prognosis in these cancers. Based on this point, SP1 has the potential to become a target to treat cancers.

The relationship between expression of SP1 and clinicopathological behaviors and prognosis of solid cancers has been addressed in many studies, so we decided to carry out this meta-analysis to evaluate the carcinogenic role of SP1 in cancers.

## Methods

### Search Strategy

This meta-analysis was performed following the convention of PRISMA (Preferred Reporting Items for Systematic Reviews and Meta-Analysis) guidelines and registered in the PROSPERO international prospective register of systematic reviews.

We extensively searched PubMed and Cochrane Library for studies published between January 1, 2000 and June 1, 2020. According to the PICO framework (population, intervention, comparison, outcome), specific search queries were formulated using terms: “SP1”, “cancer”, “clinical features” and “prognosis”. The language was limited to English.

### Selection Criteria

Primary screening of the references was performed according to title and abstract. A full-text check was then conducted in line with the inclusion and exclusion criteria.

#### Inclusion Criteria

1. Articles enrolled patients with validated histological cancer.
2. Articles were published in English.
3. Articles described SP1 and clinicopathological parameters or prognosis of cancers.

#### Exclusion Criteria

1. They were case reports, meta-analysis, reviews.
2. Studies provided insufficient data (hazard ratios with 95% confidence intervals were not available).
3. Patients accepted radiotherapy or chemotherapy before an operation.
4. Documents with a small sample size (number of patients <50).

### Articles Screening, Data Extraction, and Quality Assessment

All studies obtained from the two databases were examined by two reviewers independently. Only if both reviewers agreed to include the article, it would be included in this analysis. When any inconsistencies occurred, they would be settled by the corresponding author, who made an ultimate decision based on the original paper.

We extracted data from selected articles, including authors, year of publication, nation, antibody company, sample size and clinical features.

The Newcastle–Ottawa Quality Assessment Scale for cohort studies (NOS) was adopted to appraise the quality of every study. Every article was assessed based on patient selection, comparability of the studied group and the evaluation of the treatment outcome. In brief, a total of nine points was assigned to each research. A study whose ultimate score was more than five was considered high quality. The quality estimation was done by two authors independently. Every study got its NOS score when there were no conflicts.

### Data Analysis

This meta-analysis was conducted with *STATA* (version 15.0, StataCorp LP, College Station, TX). Basic information was provided in Table 1. We then combined all SP1 high expression groups to create a comparison with the SP1 low expression groups.

**TABLE 1:** Main characteristics and NOS scores of studies.

Author	Nation	Cancer type	Antibody company	Sample size	Median follow-up (month)	NOS score
[6]	China	Pancreatic cancer	Santa Cruz Biotechnology	80	—	8
[24]	China	Cholangiocarcinoma	Abcam	64	—	8
[10]	China	Gastric cancer	Santa Cruz Biotechnology	65	59.6	8
[19]	China	Nasopharyngeal cancer	Santa Cruz Biotechnology	82	32.4	7
[11]	China	Breast cancer	Santa Cruz Biotechnology	60	—	8
[21]	China	Pancreatic cancer	Cell Signaling Technology	77	—	8
[18]	China	Gastric cancer	Santa Cruz Biotechnology	227	—	8
[22]	China	Hepatocellular cancer	Millipore	214	13.8	7
[23]	China	Pancreatic cancer	Cell Signaling Technology	88	—	7
[25]	China	Osteosarcomas	Cell Signaling Technology	137	—	8
[30]	China	Colorectal cancer	Abcam	80	—	8
[29]	China	Esophageal cancer	Abcam	182	—	8
[28]	Korea	Pancreatic cancer	Thermo Fisher	62	—	8
[26]	China	Astrocytoma	Thermo Fisher	98	—	8
[8]	United States	Gastric cancer	Santa Cruz Biotechnology	86	25.7	7
[9]	United States	Gastric cancer	Santa Cruz Biotechnology	86	25.9	7
[13]	Korea	Gastric cancer	Santa Cruz Biotechnology	268	—	8
[12]	United States	Glioma	Santa Cruz Biotechnology	222	—	8
[15]	China	Glioma	Cell Signaling Technology	55	—	7
[14]	China	Colorectal cancer	Abcam	86	—	8
[16]	China	Breast cancer	Bio-Rad	135	—	8
[20]	China	Pancreatic cancer	Cell Signaling Technology	88	—	8
[27]	China	Esophageal cancer	Santa Cruz Biotechnology	121	—	8
[17]	China	Nasopharyngeal carcinoma	Millipore	76	—	8

NOS, Newcastle–Ottawa Quality Assessment Scale.

We used clinical experience to determine whether it was appropriate to combine trials in a meta-analysis. Odds ratios (ORs) with 95% confidence intervals (CIs) were utilized to evaluate the relevance between SP1 and clinical parameters. Hazard rations (HRs) with 95% CIs were utilized to investigate the relationship between SP1 and prognosis. Funnel plots were used to evaluate the publication bias. Heterogeneity in the studies was determined by the *I* [2] statistic (*I* [2] *>*50% suggested obvious heterogeneity). A fixed-effect model was applied when *I* [2] was less than 50%. Otherwise, we chose to use a random-effect model to perform this meta-analysis. Statistical significance was defined as *p* < 0.05. Sensitivity analysis was used to find out the source of heterogeneity. Based on the model, we assessed the correlation between SP1 expression and clinical parameters and prognosis of cancers.

### Usage of UALCAN

UALCAN (http://ualcan.path.uab.edu) was employed to investigate SP1 expression levels in different tissues, which is a free net platform to provide gene expression profiling with TCGA and clinical data and we chose box plots to realize the visualization of data [7]. Data on protein expression analysis provided by UALCAN originates from Clinical Proteomic Tumor Analysis Consortium (CPTAC) Confirmatory/Discovery dataset. Students’ t test was used to compare the expression differences and *p* < 0.05 was regarded as statistically significant. Obtained TCGA patient survival data were used for Kaplan-Meier and survival plots were adopted to assess the prognostic role of SP1. High expression means transcripts per million (TPM) value is higher than the upper quartile, and low/medium expression means TPM value is lower than the upper quartile. Log-rank *p* values display statistical significance of the patterns observed.

## Results

### Study Characteristics

**FIGURE 1:**
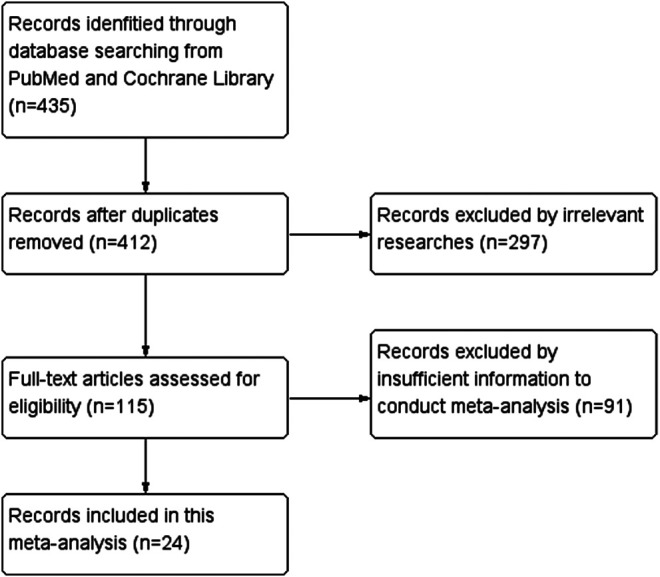
PRISMA (Preferred Reporting Items for Systematic Reviews and Meta-Analysis) flow chart.

Figure 1 is the flow chart of the essay searches and screening process for the analysis. Of 435 studies retrieved from two databases, 23 duplicates were removed, and 412 unique articles were selected. After further curation and application of the exclusion criteria, 297 irrelevant articles and 91 articles with unavailable data were excluded. Finally, 24 relevant studies reporting the association between SP1 expression and clinical indicators of cancers were included in this study.

We included 24 studies [6, 8–30] involving 2,739 patients with validated histological cancers. Table 1 summarized the baseline information (authors, year of publication, nation, cancer type, antibody company, sample size and NOS score) of each article. The included literatures came from China, United States and Korea. The participants in the studies covered a wide cancer types including pancreatic cancer, cholangiocarcinoma, gastric cancer, nasopharyngeal cancer, breast cancer, hepatocellular cancer (HCC), osteosarcomas, colorectal cancer, esophageal cancer, astrocytoma and glioma. The scores of 24 included studies range from 7 to 8, so they were all high-quality.

### Correlation of SP1 Expression With Clinicopathological Characteristics and Prognosis

**FIGURE 2:**
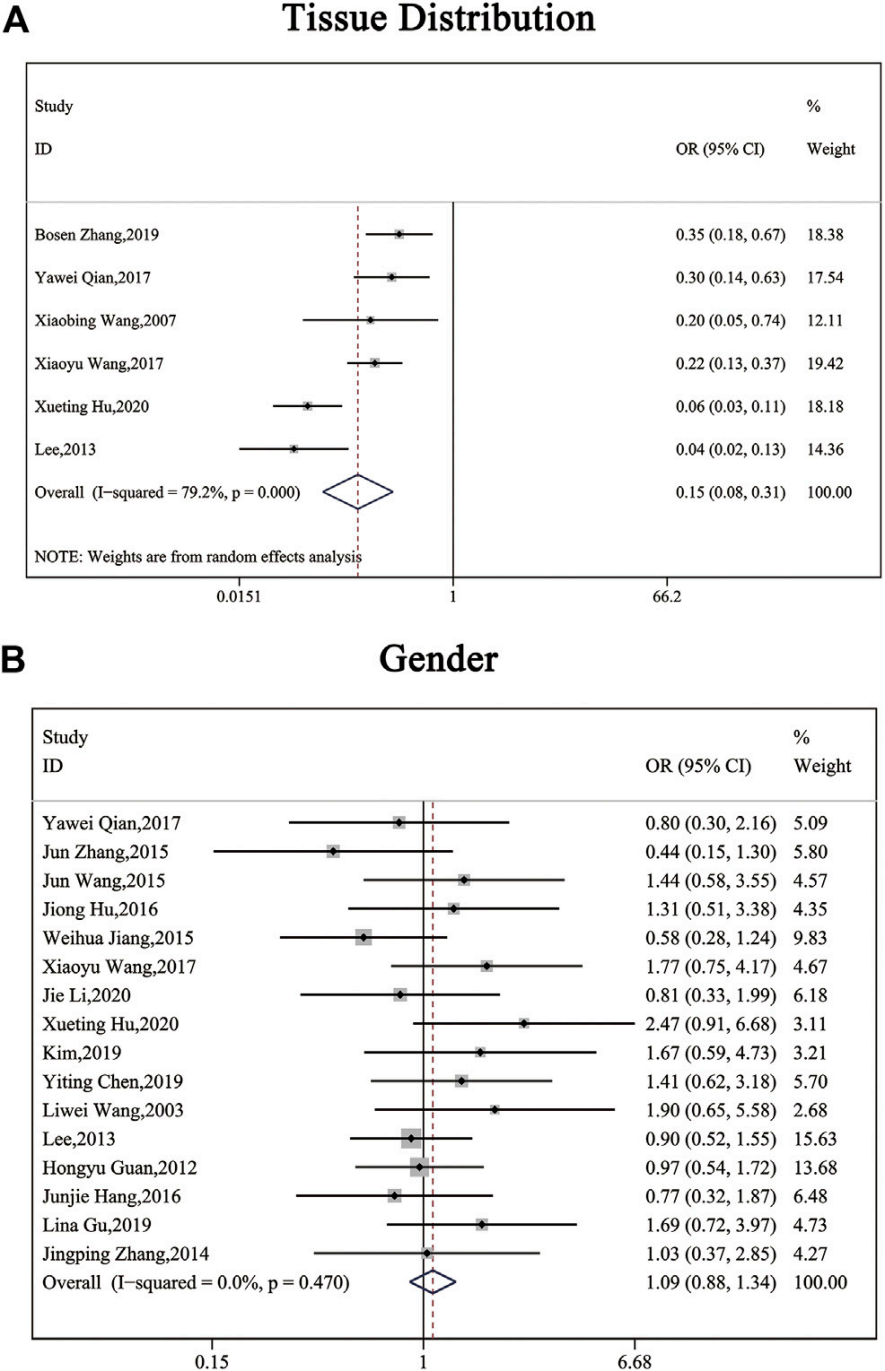
Forest plots describing the correction of SP1 expression with tissues (random-effect analysis) and gender (fixed-effect analysis). **(A)** Carcinoma tissues displayed an elevated SP1 expression. **(B)** SP1 expression was independent of gender. Abbreviations: OR, odds ratio; CI, confidence interval.

**FIGURE 3:**
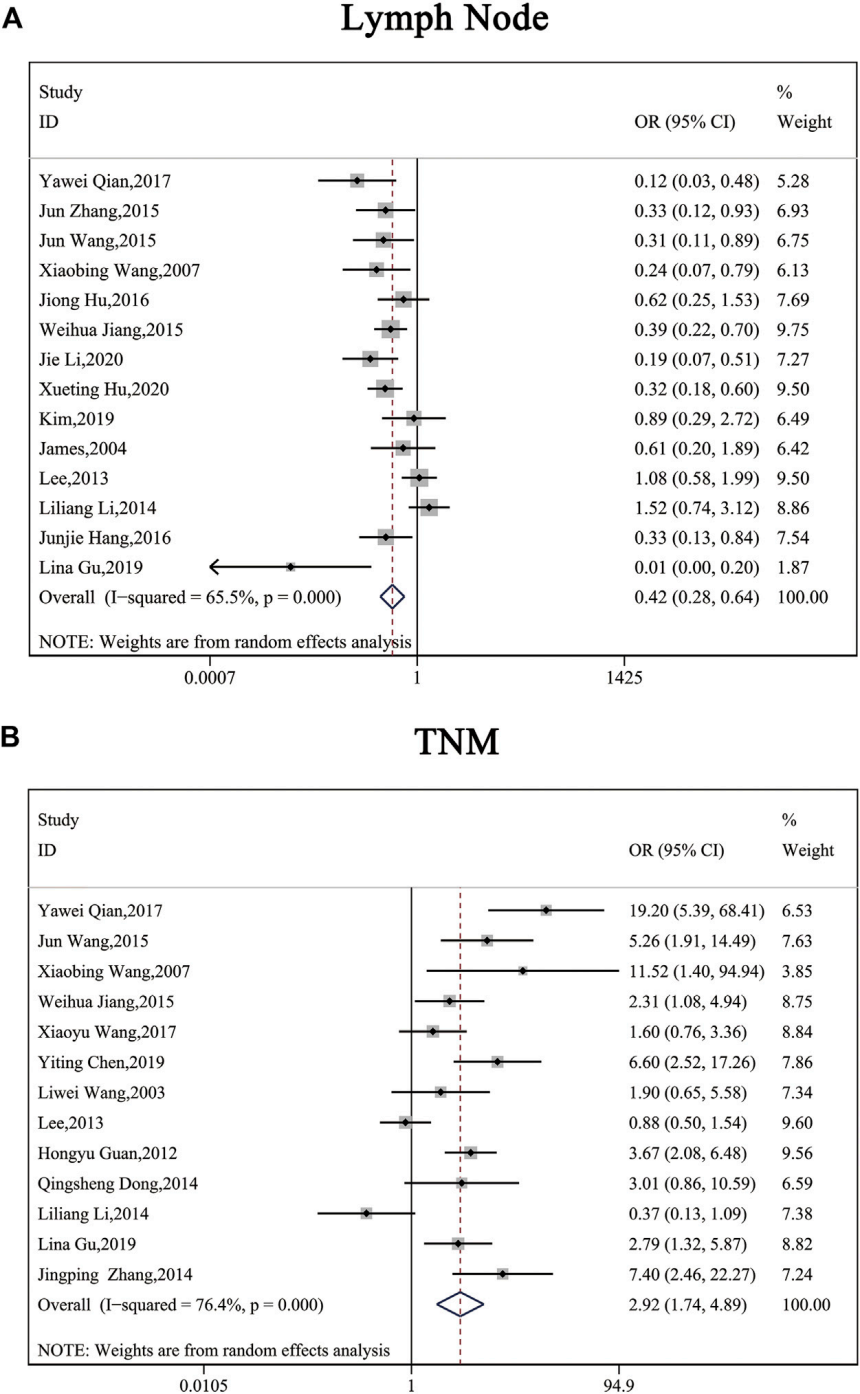
Forest plots describing the correction of SP1 expression with lymph node metastasis (random-effect analysis) and TNM stage (random-effect analysis). **(A)** High SP1 expression promoted lymph node metastasis. **(B)** SP1 expression was associated with TNM stage. Abbreviations: OR, odds ratio; CI, confidence interval.

The clinical parameters of studies were listed in Table 2. A total of six articles, including 1,304 patients with pancreatic cancer, cholangiocarcinoma, breast cancer, osteosarcomas, esophageal cancer or gastric cancer, investigated SP1 expression in tissues. Adjacent normal tissues had a lower level of SP1 (OR = 0.15; 95%CI:0.08-0.31; *p* < 0.05) in comparison with tumor sites (Figure 1A). In addition, SP1 expression was linked with lymph node metastasis (OR = 0.42; 95%CI:0.28-0.64; *p* < 0.05) (Figure 2A), advanced TNM stage (OR = 0.34; 95%CI:0.20-0.57; *p* < 0.05) (Figure 2B) and infiltration depth (OR = 0.33; 95%CI:0.18-0.60; *p* < 0.05) (Figure 3A) which was independent of gender (OR = 1.09; 95%CI:0.88-1.34; *p* > 0.05) (Figure 1B). A total of seven studies enrolling 951 patients surveyed survival data (Table 3). We concluded that enhanced SP1 expression was positively associated with unsatisfactory prognosis of various types of cancers including HCC, gastric cancer, pancreatic cancer and esophageal cancer (HR = 1.95; 95%CI:1.16-3.28; *p* < 0.05) (Figure 3B).

**TABLE 2:** Extracted data of clinicopathological parameters from included studies.

Author	Gender				Tissue				Lymph node metastasis				TNM Stage				Infiltration				
	Male		Female		Cancer		Normal		Yes		No		I–II		III–IV		T1–T2		T3–T4		
	+	−	+	−	+	−	+	−	+	−	+	−	+	−	+	−	+	−	+	−
[6]	N	N	N	N	57	23	37	43	N	N	N	N	N	N	N	N	N	N	N	N
[24]	20	15	15	14	35	29	17	47	17	3	18	26	7	24	28	5	N	N	N	N
[10]	27	18	8	12	N	N	N	N	21	11	14	19	N	N	N	N	8	19	27	11
[19]	23	29	16	14	N	N	N	N	33	27	6	16	7	23	32	20	11	29	28	14
[11]	N	N	N	N	43	17	4	8	32	7	11	10	25	16	18	1	N	N	N	N
[21]	24	27	14	12	N	N	N	N	23	19	15	20	N	N	N	N	N	N	N	N
[18]	137	20	56	14	N	N	N	N	117	14	76	20	81	22	102	12	N	N	N	N
[22]	N	N	N	N	N	N	N	N	N	N	N	N	N	N	N	N	N	N	N	N
[23]	N	N	N	N	N	N	N	N	N	N	N	N	N	N	N	N	N	N	N	N
[25]	55	54	18	10	73	64	27	110	N	N	N	N	42	55	22	18	N	N	N	N
[30]	17	15	23	25	N	N	N	N	24	9	16	31	N	N	N	N	13	32	27	8
[29]	81	80	15	6	96	86	11	171	69	39	27	47	N	N	N	N	33	39	63	47
[28]	9	19	15	19	N	N	N	N	17	26	7	12	N	N	N	N	19	32	5	6
[26]	30	26	26	16	N	N	N	N	N	N	N	N	14	22	42	20	N	N	N	N
[8]	22	8	47	9	N	N	N	N	N	N	N	N	22	8	47	9	N	N	N	N
[9]	N	N	N	N	N	N	N	N	46	13	23	4	N	N	N	N	N	N	N	N
[13]	43	120	75	30	195	73	4	34	132	51	53	19	75	26	119	47	76	26	115	46
[12]	90	63	40	29	N	N	N	N	N	N	N	N	34	52	96	40	N	N	N	N
[15]	N	N	N	N	N	N	N	N	N	N	N	N	8	7	31	9	N	N	N	N
[14]	N	N	N	N	N	N	N	N	N	N	N	N	N	N	N	N	3	8	58	17
[16]	N	N	N	N	N	N	N	N	31	15	83	6	52	5	62	16	N	N	N	N
[20]	35	21	18	14	N	N	N	N	27	9	26	26	N	N	N	N	43	31	10	4
[27]	41	52	16	12	N	N	N	N	57	37	0	27	18	36	39	28	N	N	N	N
[17]	31	24	12	9	N	N	N	N	N	N	N	N	6	18	37	15	13	22	30	11

N, not reported.

**TABLE 3:** Extracted data of prognosis from included studies.

Author	Cancer type	Hazard ratio	95% Confidence interval
[22]	Hepatocellular cancer	1.907	1.067–3.408
[23]	Pancreatic cancer	2.27	1.24–4.16
[8]	Gastric cancer	4.5	1.8–11.2
[9]	Gastric cancer	3.54	1.2–10.42
[13]	Gastric cancer	0.603	0.346–1.151
[20]	Pancreatic cancer	4.48	1.14–17.62
[27]	Esophageal cancer	1.281	0.776–2.114

### Publication Bias

**FIGURE 4:**
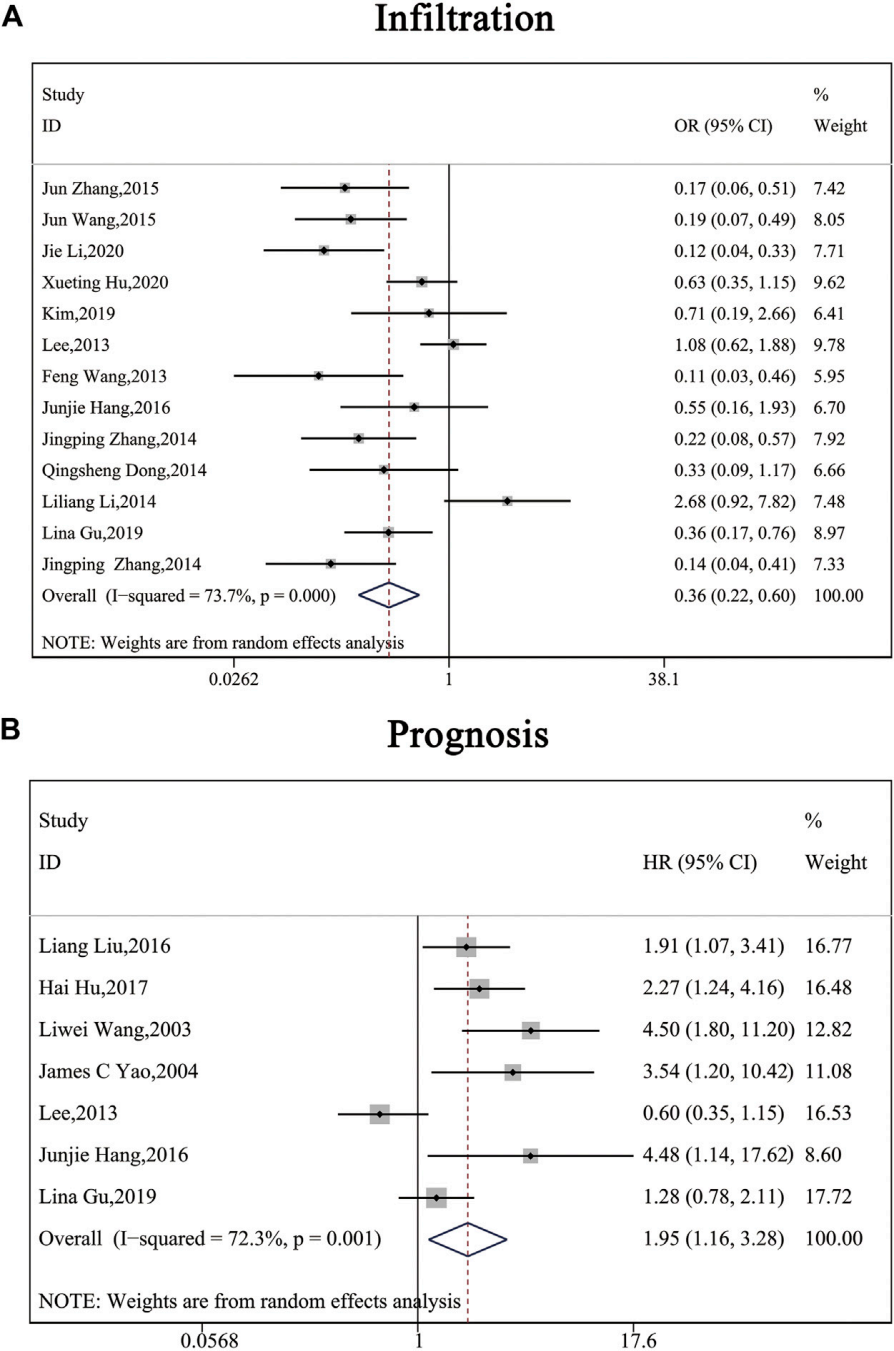
Forest plots describing the correction of SP1 expression with depth of invasion (random-effect analysis) and prognosis (random-effect analysis). **(A)** SP1 expression influenced depth of invasion. **(B)** High SP1 expression related to poor prognosis. Abbreviations: OR, odds ratio; CI, confidence interval; HR, hazard ratio.

Funnel plots with pseudo 95% confidence limits were applied to examine publication bias, as illustrated in Figure 4. Evidence showed the funnel plots for tissue distribution, gender, lymph node metastasis, TNM stage, infiltration and prognosis were symmetrical, so there was no apparent risk of bias. This indicated the results of this meta-analysis were reliable.

### Heterogeneity and Sensitivity Analysis

**FIGURE 5:**
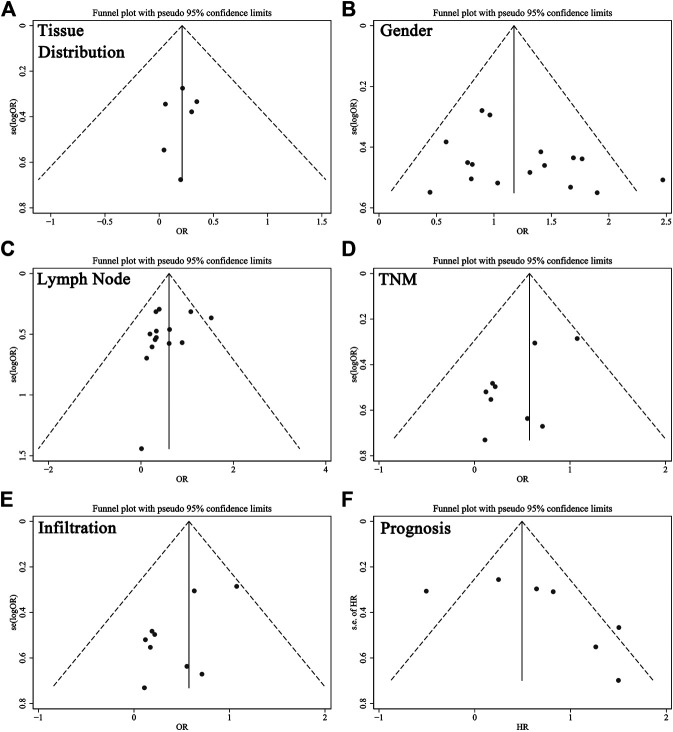
Funnel plots for the evaluation of potential publication bias about **(A)** tissues, **(B)** gender, **(C)** lymph node metastasis, **(D)** TNM stage, **(E)** infiltration and **(F)** prognosis. Abbreviations: OR, odds ratio; HR, hazard ratio.

Because of the high level of heterogeneity (*I* ([2])>50%), random-effect models were used on publications about tissue distribution, lymph node metastasis, TNM stage, infiltration and prognosis. We adopted a sensitivity analysis to define whether the exclusion of an individual document influenced overall results (Figure 5). Evidence showed that no matter which separate study was screened out of this analysis, the aggregate estimate of the effect of SP1 expression level on tissue distribution, lymph node metastasis, TNM stage, infiltration and survival time had no significant change.

### Bioinformatics Analysis

**FIGURE 6:**
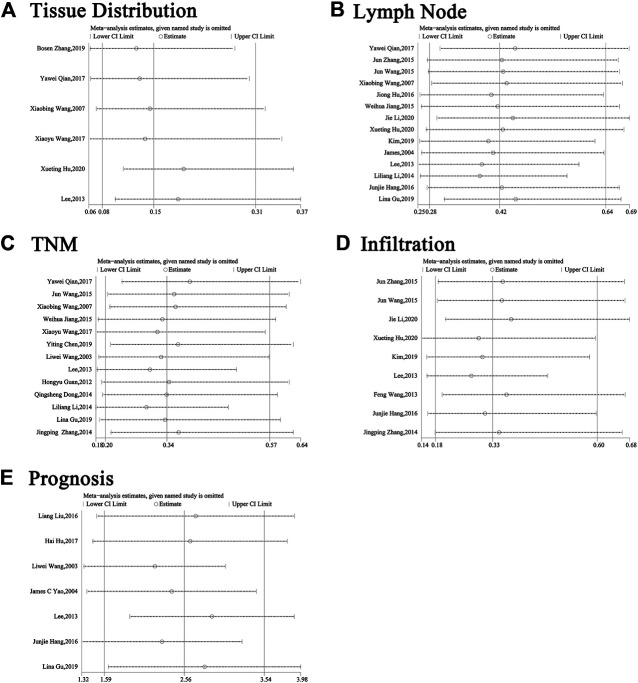
Sensitivity analysis about **(A)** tissues, **(B)** gender, **(C)** lymph node metastasis, **(D)** TNM stage, **(E)** infiltration and **(F)** prognosis. Abbreviations: CI, confidence interval.

**FIGURE 7:**
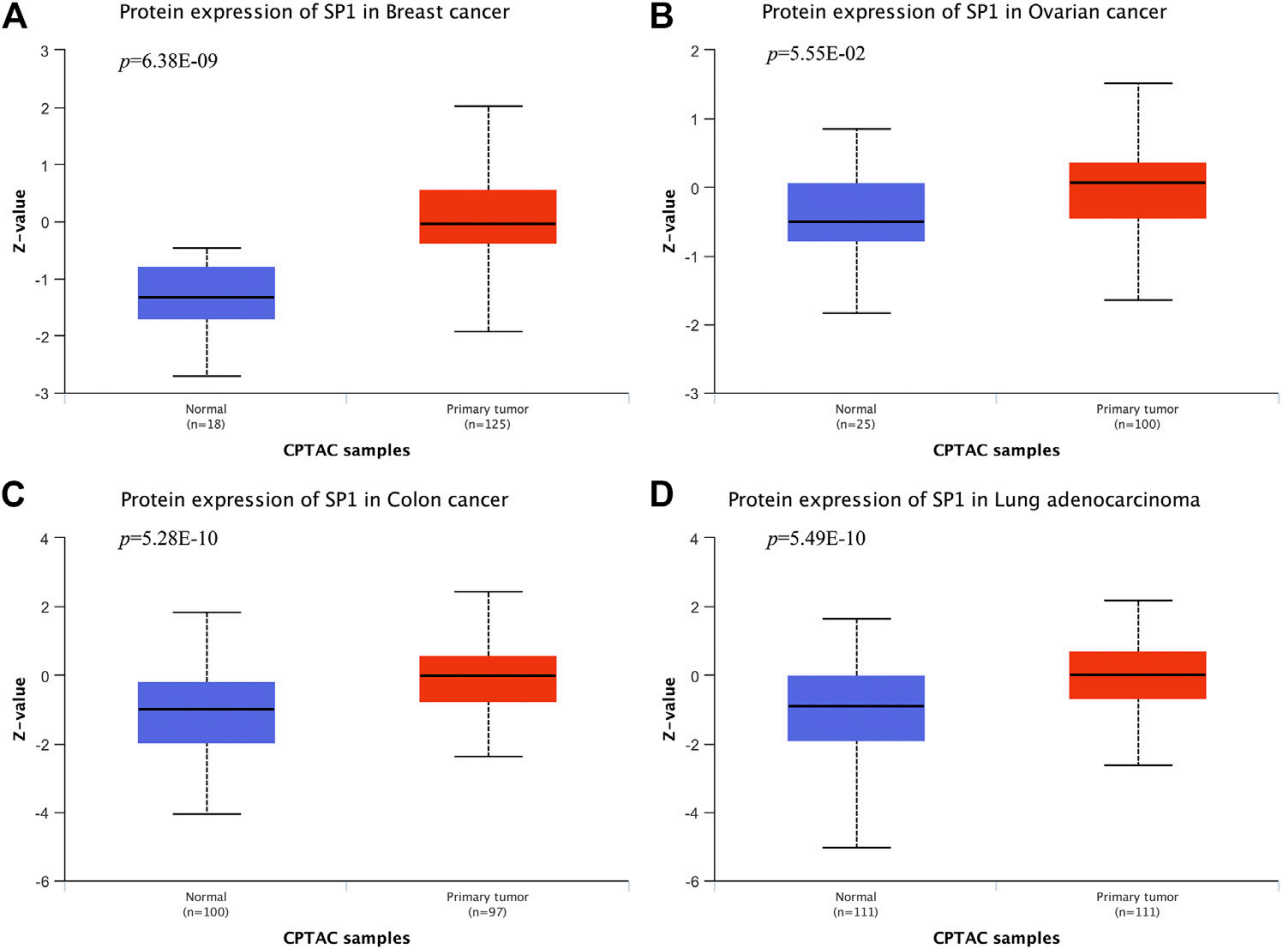
SP1 protein expression in cancers (UALCAN). SP1 displayed an increased expression level in **(A)** breast cancer, **(B)** ovarian cancer, **(C)** colon cancer and **(D)** lung adenocarcinoma.

According to CPTAC dataset from UALCAN, protein expression of SP1 was higher in breast cancer, ovarian cancer, colon cancer and lung cancer, compared with their corresponding normal tissues (Figure 6), and the difference was significant. Kaplan-Meier plots showed the association between SP1 transcription expression and survival time. Although the relevance of SP1 mRNA level with the prognosis of breast cancer and colon adenocarcinoma was not statistically significant, the overall tendency was distinct (Figure 7). Furthermore, enhanced SP1 mRNA level tended to result in a poor clinical outcome of liver hepatocellular carcinoma and brain lower grade glioma, suggesting its vital role in tumorigenesis. However, more thorough researches are essential to validate the correlation of SP1 with tumor prognosis.

**FIGURE 8:**
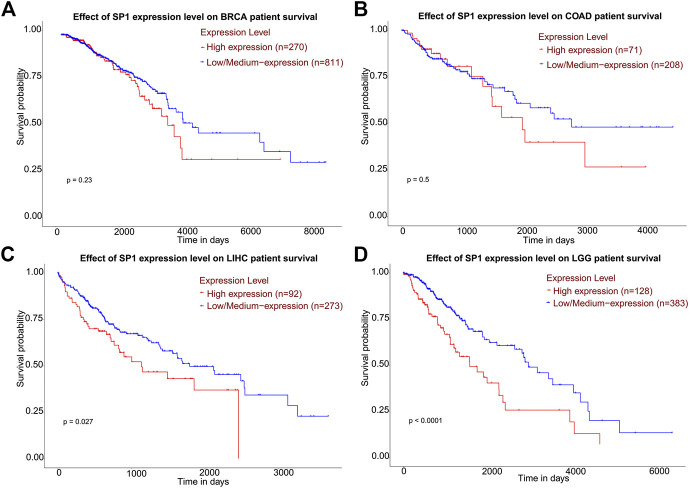
Kaplan-Meier plots describing gene-level correlations with patient survival (UALCAN). The correlation of SP1 expression with prognosis of **(A)** breast invasive carcinoma and **(B)** colon adenocarcinoma was not statistically significant. High SP1 expression was associated with shorter survival of **(C)** liver hepatocellular carcinoma and **(D)** brain lower grade glioma. Abbreviations: BRCA, breast invasive carcinoma; COAD, colon adenocarcinoma; LIHC, liver hepatocellular carcinoma; LGG, brain lower grade glioma.

## Discussion

Herein, we retrieved all available publications and conducted this meta-analysis to clarify the clinical significance of SP1. A total of 24 articles enrolling 2,739 patients with solid tumors confirmed by histopathology were selected in our review. Some clinical and pathological characteristics were observed such as infiltration depth, lymph node metastasis and TNM stage. Our review revealed that SP1 showed a higher level in pancreatic cancer, cholangiocarcinoma, breast cancer, osteosarcomas, esophageal cancer and gastric cancer (OR = 0.15; 95% CI: 0.08-0.31; *p* < 0.05), and lower SP1 was detrimental to lymph node migration (OR = 0.42; 95% CI: 0.28-0.64; *p* < 0.05), advance of TNM stage (OR = 0.34; 95% CI: 0.20-0.57; *p* < 0.05) and exacerbation of infiltration (OR = 0.33; 95% CI: 0.18-0.60; *p* < 0.05). In HCC, gastric cancer, pancreatic cancer and esophageal cancer, patients with increased SP1 were more likely to have poor outcomes (HR = 1.95; 95% CI: 1.16-3.28; *p* < 0.05). Funnel plots disclosed there was no significant publication bias. The year of publication, sample size and nation may be the reasons for the high level of heterogeneity. Sensitivity analysis showed the alterations of selected criteria did not change the pooled results. Bioinformatics analysis discovered differentially expressed SP1 might influence the prognosis of tumors. Our paper was of great significance since it uncovered the clinical significance of SP1 for cancer patients.

SP1 regulates tumorigenesis via enhancing transcription of downstream genes whose promoter contain CG-rich typical sequence, resulting in a poor prognosis of patients [31]. Liu *et al* demonstrated SP1 bound Annexin A2 promoter to activate its transcription, subsequently promoting the metastasis and invasion of oral squamous cell carcinoma [32]. FoxO3a is also a transcription regulator which plays a crucial role in carcinogenesis. Yu *et al.* [33] found FoxO3a promoter had a large amount of potential SP1 binding sites, and they confirmed that SP1-contributed transcription of FoxO3a facilitated colorectal cancer progression. In HCC, SP1 not only activates transcription of metastasis-associated lung adenocarcinoma transcript 1 (MALAT1), but also relates to AFP level, implying it can be a novel marker in HCC screening [34].

SP1 relates to Wnt signaling pathway and they can maintain the stability of each other. When Wnt signaling is off, SP1 constancy is destroyed by glycogen synthase 3β (GSK3β)-induced phosphorylation and β-TrCP E3 ubiquitin ligase-induced ubiquitination. When Wnt signaling is switched on, the interrelation of SP1 with elements of the destruction complex is blocked, avoiding consecutive degradation of SP1. On the other hand, chromatin immunoprecipitation sequencing (ChIP-seq) verifies SP1 controls β-catenin steadiness and regulates Wnt-related genes [35]. This indicates SP1 and Wnt pathway form a positive feedback loop to boost the progression of cancers. In breast cancer, SP1 stimulates non-classical Wnt response gene subgroups to shorten the overall survival of patients [36]. Cytochrome P450 1B1 (CYP1B1) is reported to elevate in breast cancer and prostate cancer. It exerts carcinogenic effects via EMT and initiation of Wnt signaling pathway and mechanistic analysis reveals the process is mediated by SP1 [37]. A similar interplay of SP1 and Wnt signal is also observed in prostate cancer [38].

Estrogen receptor (ER) acts as a prognostic factor of breast cancer, determining the response to endocrine therapy. According to Cheang, SP1 is more sensitive than 1D5 in identifying patients with satisfactory outcomes among those with ER-positive breast carcinomas [39]. Similarly, SP1 is also superior to 1D5 for authenticating ER status, which demonstrated SP1 has advantages in becoming biomarkers [40]. Young *et al* agreed with this statement, holding that SP1 was the most sensitive antibody to identify ER expression in breast cancer [41]. Huang *et al* confirmed that in HCC, SP1 had a correlation with AFP level (*r* = 7.44, *p* = 0.0064) [34]. In lung cancer tissues, the positive rate of SP1 was found to be higher than that of APF [42]. NG Ordonez reported that higher sensitivity can be obtained in distinguishing between malignant epithelial pleural mesothelioma and lung adenocarcinoma by immunostaining CEA and SP1 [43]. What’s more, the AUC of the combination of SP1/SP3/FLIP with Gleason score for predicting PSA failure and non-failure was 0.93 [44]. The results highlight the possibility of SP1 becoming a clinical target of some specific cancers.

Accumulating proof has shown SP1 expression correlates with proliferation, migration, invasion and cell cycle in cancer cells. Zhao et al. [45] observed deregulation of SP1 in HCC, which affected the growth of liver cancer cells, suggesting SP1 would become a target in HCC treatment. Additionally, SP1 was directly targeted by miR-382, a tumor suppressor gene, impeding colorectal cancer cell development and metastasis [46]. Wang et al. [47] reported SP1 regulated gastric cancer cell hyperplasia and cell cycle in a UCA1-dependent manner, supporting its important role in various cancers.

Our review had a lot of strengths. Firstly, we performed a meta-analysis and bioinformatics analysis to support our results. Secondly, The Newcastle–Ottawa Quality Assessment Scale was adopted to evaluate the quality of included studies and all studies were considered high-quality. Furthermore, it was the first to verify SP1 correlated with clinicopathological parameters and poor prognosis in neoplasm.

Our review also had some limitations. To start with, although we searched PubMed and Cochrane Library exhaustively, the number of included literatures is still very small, which prevented us from conducting subgroup analysis based on tumor type. Then, most of the documents we selected were from China. However, we had strict screening criteria and all studies were regarded as high-quality. Finally, data on prognosis and the relative expression of SP1 in tissues was relatively little so we could not absolutely say SP1 was an oncogene in tumors.

In all, carcinoma tissues tended to have elevated SP1 expression, which is related to clinicopathological characteristics of tumors, implying the central role of SP1 in tumor progression. However, due to the limitations we mentioned above, the results must be explained carefully and more clinical trials of SP1 in human solid tumors are required for a more reliable conclusion.

## Conclusion

SP1 expression is connected with lymph node metastasis, TNM stage and infiltration depth, suggesting it can be considered as a potential target for cancer treatment in the future.

## Data Availability

The raw data supporting the conclusions of this article will be made available by the authors, without undue reservation.

## Abbreviations

BRCA, Breast Invasive Carcinoma; ChIP-seq, Chromatin immunoprecipitation sequencing; CI, Confidence interval; COAD, Colon Adenocarcinoma; CPTAC, Clinical Proteomic Tumor Analysis Consortium; CYP1B1, Cytochrome P450 1B1; EMT, Epithelial-mesenchymal transformation; ER, estrogen receptor; GSK3β, Glycogen synthase 3β; HCC, Hepatocellular carcinoma; HR, Hazard ratio IGFIR Insulin-like growth factors I receptor; LGG, Brain Lower Grade Glioma; LIHC, Liver Hepatocellular Carcinoma; MALAT1, Metastasis-associated lung adenocarcinoma transcript 1; MMP-2, Matrix metalloproteinase-2; MMP-9, Matrix metalloproteinase-9; NOS, Newcastle–Ottawa Quality Assessment Scale; OR, Odds ratio; PRISMA, Preferred Reporting Items for Systematic Reviews and Meta-Analysis; SP1, Specificity protein 1; TPM, Transcripts per million; VEGF, Vascular endothelial growth factor.

## References

[1] Beishline K. Azizkhan-Clifford J. (2015). Sp1 and the ‘hallmarks of cancer’. FEBS J. 282 (2), 224–258. 10.1111/febs.13148 25393971

[2] Maor S. Mayer D. Yarden R. I. Lee A. V. Sarfstein R. Werner H. (2006). Estrogen receptor regulates insulin-like growth factor-I receptor gene expression in breast tumor cells: involvement of transcription factor Sp1. J. Endocrinol. 191 (3), 605–612. 10.1677/joe.1.07016 17170218

[3] Santra M. Santra S. Zhang J. Chopp M. (2008). Ectopic decorin expression up-regulates VEGF expression in mouse cerebral endothelial cells via activation of the transcription factors Sp1, HIF1alpha, and Stat3. J. Neurochem. 105 (2), 324–337. 10.1111/j.1471-4159.2007.05134.x 18021292

[4] Hung W. C. Tseng W. L. Shiea J. Chang H. C. (2010). Skp2 overexpression increases the expression of MMP-2 and MMP-9 and invasion of lung cancer cells. Canc. Lett. 288 (2), 156–161. 10.1016/j.canlet.2009.06.032 19625121

[5] Chang S. Sun L. Feng G. (2019). SP1-mediated long noncoding RNA POU3F3 accelerates the cervical cancer through miR-127-5p/FOXD1. Biomed. Pharmacother. 117, 109133. 10.1016/j.biopha.2019.109133 31252264

[6] Zhang B. Song L. Cai J. Li L. Xu H. Li M. (2019). The LIM protein Ajuba/SP1 complex forms a feed forward loop to induce SP1 target genes and promote pancreatic cancer cell proliferation. J. Exp. Clin. Canc. Res. 38 (1), 205. 10.1186/s13046-019-1203-2 PMC6525466 31101117

[7] Chandrashekar D. S. Bashel B. Balasubramanya S. A. H. Creighton C. J. Ponce-Rodriguez I. Chakravarthi B. V. S. K. (2017). UALCAN: a portal for facilitating tumor subgroup gene expression and survival analyses. Neoplasia. 19 (8), 649–658. 10.1016/j.neo.2017.05.002 28732212 PMC5516091

[8] Wang L. Wei D. Huang S. Peng Z. Le X. Wu T. T. (2003). Transcription factor Sp1 expression is a significant predictor of survival in human gastric cancer. Clin. Canc. Res. 9 (17), 6371–6380. 14695137

[9] Yao J. C. Wang L. Wei D. Gong W. Hassan M. Wu T. T. (2004). Association between expression of transcription factor Sp1 and increased vascular endothelial growth factor expression, advanced stage, and poor survival in patients with resected gastric cancer. Clin. Canc. Res. 10 (12 Pt 1), 4109–4117. 10.1158/1078-0432.CCR-03-0628 15217947

[10] Zhang J. Zhu Z. G. Ji J. Yuan F. Yu Y. Y. Liu B. Y. (2005). Transcription factor Sp1 expression in gastric cancer and its relationship to long-term prognosis. World J. Gastroenterol. 11 (15), 2213–2217. 10.3748/wjg.v11.i15.2213 15818728 PMC4305801

[11] Wang X. B. Peng W. Q. Yi Z. J. Zhu S. L. Gan Q. H. (2007). [Expression and prognostic value of transcriptional factor sp1 in breast cancer]. Ai Zheng. 26 (9), 996–1000. 17927860

[12] Guan H. Cai J. Zhang N. Wu J. Yuan J. Li J. (2012). Sp1 is upregulated in human glioma, promotes MMP-2-mediated cell invasion and predicts poor clinical outcome. Int. J. Canc. 130 (3), 593–601. 10.1002/ijc.26049 21469139

[13] Lee H. S. Park C. K. Oh E. Erkin Ö. C. Jung H. S. Cho M. H. (2013). Low SP1 expression differentially affects intestinal-type compared with diffuse-type gastric adenocarcinoma. PloS One. 8 (2), e55522. 10.1371/journal.pone.0055522 23437057 PMC3577840

[14] Wang F. Ma Y. L. Zhang P. Shen T. Y. Shi C. Z. Yang Y. Z. (2013). SP1 mediates the link between methylation of the tumour suppressor miR-149 and outcome in colorectal cancer. J. Pathol. 229 (1), 12–24. 10.1002/path.4078 22821729

[15] Dong Q. Cai N. Tao T. Zhang R. Yan W. Li R. (2014). An axis involving SNAI1, microRNA-128 and SP1 modulates glioma progression. PloS One. 9 (6), e98651. 10.1371/journal.pone.0098651 24959930 PMC4068992

[16] Li L. Gao P. Li Y. Shen Y. Xie J. Sun D. (2014). JMJD2A-dependent silencing of Sp1 in advanced breast cancer promotes metastasis by downregulation of DIRAS3. Breast Canc. Res. Treat. 147 (3), 487–500. 10.1007/s10549-014-3083-7 25193278

[17] Zhang J. P. Zhang H. Wang H. B. Li Y. X. Liu G. H. Xing S. (2014). Down-regulation of Sp1 suppresses cell proliferation, clonogenicity and the expressions of stem cell markers in nasopharyngeal carcinoma. J. Transl. Med. 12, 222. 10.1186/s12967-014-0222-1 25099028 PMC4132216

[18] Jiang W. Jin Z. Zhou F. Cui J. Wang L. Wang L. (2015). High co-expression of Sp1 and HER-2 is correlated with poor prognosis of gastric cancer patients. Surg. Oncol. 24 (3), 220–225. 10.1016/j.suronc.2015.05.004 26096373

[19] Wang J. Kang M. Qin Y. T. Wei Z. X. Xiao J. J. Wang R. S. (2015). Sp1 is over-expressed in nasopharyngeal cancer and is a poor prognostic indicator for patients receiving radiotherapy. Int. J. Clin. Exp. Pathol. 8 (6), 6936–6943. 26261581 PMC4525915

[20] Hang J. Hu H. Huang J. Han T. Zhuo M. Zhou Y. (2016). Sp1 and COX2 expression is positively correlated with a poor prognosis in pancreatic ductal adenocarcinoma. Oncotarget. 7 (19), 28207–28217. 10.18632/oncotarget.8593 27057636 PMC5053721

[21] Hu J. Hu H. Hang J. J. Yang H. Y. Wang Z. Y. Wang L. (2016). Simultaneous high expression of PLD1 and Sp1 predicts a poor prognosis for pancreatic ductal adenocarcinoma patients. Oncotarget. 7 (48), 78557–78565. 10.18632/oncotarget.12447 27713167 PMC5346659

[22] Liu L. Ji P. Qu N. Pu W. L. Jiang D. W. Liu W. Y. (2016). The impact of high co-expression of Sp1 and HIF1α on prognosis of patients with hepatocellular cancer. Oncol. Lett. 12 (1), 504–512. 10.3892/ol.2016.4634 27347172 PMC4906840

[23] Hu H. Wu L. L. Han T. Zhuo M. Lei W. Cui J. J. (2017). Correlated high expression of FXR and Sp1 in cancer cells confers a poor prognosis for pancreatic cancer: a study based on TCGA and tissue microarray. Oncotarget. 8 (20), 33265–33275. 10.18632/oncotarget.16633 28402278 PMC5464866

[24] Qian Y. Yao W. Yang T. Yang Y. Liu Y. Shen Q. (2017). aPKC-ι/P-Sp1/Snail signaling induces epithelial-mesenchymal transition and immunosuppression in cholangiocarcinoma. Hepatology. 66 (4), 1165–1182. 10.1002/hep.29296 28574228

[25] Wang X. X. Liao Y. Hong L. Zeng Z. Yuan T. B. Xia X. (2017). Tissue microarray staining reveals PLD1 and Sp1 have a collaborative, pro-tumoral effect in patients with osteosarcomas. Oncotarget. 8 (43), 74340–74347. 10.18632/oncotarget.20605 29088790 PMC5650345

[26] Chen Y. T. Tsai H. P. Wu C. C. Chen C. Y. Chai C. Y. Kwan A. L. (2019). High-level Sp1 is associated with proliferation, invasion, and poor prognosis in astrocytoma. Pathol. Oncol. Res. 25 (3), 1003–1013. 10.1007/s12253-018-0422-8 29948615

[27] Gu L. Sang M. Li J. Liu F. Wu Y. Liu S. (2019). Expression and prognostic significance of MAGE-A11 and transcription factors (SP1,TFCP2 and ZEB1) in ESCC tissues. Pathol. Res. Pract. 215 (7), 152446. 10.1016/j.prp.2019.152446 31126819

[28] Kim I. K. Lee Y. S. Kim H. S. Dong S. M. Park J. S. Yoon D. S. (2019). Specific protein 1(SP1) regulates the epithelial-mesenchymal transition via lysyl oxidase-like 2(LOXL2) in pancreatic ductal adenocarcinoma. Sci. Rep. 9 (1), 5933. 10.1038/s41598-019-42501-6 30976063 PMC6459819

[29] Hu X. Lin J. Jiang M. He X. Wang K. Wang W. (2020). HIF-1α promotes the metastasis of esophageal squamous cell carcinoma by targeting SP1. J. Canc. 11 (1), 229–240. 10.7150/jca.35537 PMC6930417 31892989

[30] Li J. Peng W. Yang P. Chen R. Gu Q. Qian W. (2020). MicroRNA-1224-5p inhibits metastasis and epithelial-mesenchymal transition in colorectal cancer by targeting SP1-mediated NF-κB signaling pathways. Front. Oncol. 10, 294. 10.3389/fonc.2020.00294 32231999 PMC7083241

[31] Bajpai R. Nagaraju G. P. (2017). Specificity protein 1: its role in colorectal cancer progression and metastasis. Crit. Rev. Oncol. Hematol. 113, 1–7. 10.1016/j.critrevonc.2017.02.024 28427500

[32] Liu X. B. Wang J. Li K. Fan X. N. (2019). Sp1 promotes cell migration and invasion in oral squamous cell carcinoma by upregulating Annexin A2 transcription. Mol. Cell. Probes. 46, 101417. 10.1016/j.mcp.2019.06.007 31254619

[33] Yu Y. Peng K. Li H. Zhuang R. Wang Y. Li W. (2018). SP1 upregulated FoxO3a promotes tumor progression in colorectal cancer. Oncol. Rep. 39 (5), 2235–2242. 10.3892/or.2018.6323 29565456

[34] Huang Z. Huang L. Shen S. Li J. Lu H. Mo W. (2015). Sp1 cooperates with Sp3 to upregulate MALAT1 expression in human hepatocellular carcinoma. Oncol. Rep. 34 (5), 2403–2412. 10.3892/or.2015.4259 26352013

[35] Mir R. Sharma A. Pradhan S. J. Galande S. (2018). Regulation of transcription factor SP1 by the β-catenin destruction complex modulates Wnt response. Mol. Cell Biol. 38 (22), e00188-18. 10.1128/MCB.00188-18 30181396 PMC6206460

[36] Monteleone E. Orecchia V. Corrieri P. Schiavone D. Avalle L. Moiso E. (2019). SP1 and STAT3 functionally synergize to induce the RhoU small GTPase and a subclass of non-canonical WNT responsive genes correlating with poor prognosis in breast cancer. Cancers. 11 (1), 101. 10.3390/cancers11010101 PMC6356433 30654518

[37] Kwon Y. J. Baek H. S. Ye D. J. Shin S. Kim D. Chun Y. J. (2016). CYP1B1 enhances cell proliferation and metastasis through induction of EMT and activation of wnt/β-catenin signaling via Sp1 upregulation. PloS One. 11 (3), e0151598. 10.1371/journal.pone.0151598 26981862 PMC4794175

[38] Sun Y. Xu K. He M. Fan G. Lu H. (2018). Overexpression of glypican 5 (GPC5) inhibits prostate cancer cell proliferation and invasion via suppressing sp1-mediated EMT and activation of wnt/β-catenin signaling. Oncol. Res. 26 (4), 565–572. 10.3727/096504017X15044461944385 28893348 PMC7844840

[39] Cheang M. C. Treaba D. O. Speers C. H. Olivotto I. A. Bajdik C. D. Chia S. K. (2006). Immunohistochemical detection using the new rabbit monoclonal antibody SP1 of estrogen receptor in breast cancer is superior to mouse monoclonal antibody 1D5 in predicting survival. J. Clin. Oncol. 24 (36), 5637–5644. 10.1200/JCO.2005.05.4155 17116944

[40] Welsh A. W. Harigopal M. Wimberly H. Prasad M. Rimm D. L. (2013). Quantitative analysis of estrogen receptor expression shows SP1 antibody is more sensitive than 1D5. Appl. Immunohistochem. Mol. Morphol. 21 (2), 139–147. 10.1097/PAI.0b013e31825d73b2 22820659 PMC3482297

[41] Bae Y. K. Gong G. Kang J. Lee A. Cho E. Y. Lee J. S. (2012). Hormone receptor expression in invasive breast cancer among Korean women and comparison of 3 antiestrogen receptor antibodies: a multi-institutional retrospective study using tissue microarrays. Am. J. Surg. Pathol. 36 (12), 1817–1825. 10.1097/PAS.0b013e318267b012 23154769

[42] Kozlowicz-Gudzinska I. Pietraszek A. Kaminska J. (1989). Initial concentration of the neoplastic biological markers AFP (alpha fetoprotein), HCG (chorionic gonadotropin), and SP1 (specific pregnancy protein) in separate types and stages of clinically advanced lung cancer]. Nowotwory. 39 (3-4), 162–165. 2484017

[43] Ordonez N. G. (1989). The immunohistochemical diagnosis of mesothelioma. Differentiation of mesothelioma and lung adenocarcinoma. Am. J. Surg. Pathol. 13 (4), 276–291. 2648877

[44] Bedolla R. G. Gong J. Prihoda T. J. Yeh I. T. Thompson I. M. Ghosh R. (2012). Predictive value of Sp1/Sp3/FLIP signature for prostate cancer recurrence. PloS One. 7 (9), e44917. 10.1371/journal.pone.0044917 23028678 PMC3441693

[45] Zhao Q. Cai W. Zhang X. Tian S. Zhang J. Li H. (2017). RYBP expression is regulated by KLF4 and Sp1 and is related to hepatocellular carcinoma prognosis. J. Biol. Chem. 292 (6), 2143–2158. 10.1074/jbc.M116.770727 28028181 PMC5313089

[46] Ren Y. Zhang H. Jiang P. (2018). MicroRNA-382 inhibits cell growth and migration in colorectal cancer by targeting SP1. Biol. Res. 51 (1), 51. 10.1186/s40659-018-0200-9 30474556 PMC6260849

[47] Wang Z. Q. Cai Q. Hu L. He C. Y. Li J. F. Quan Z. W. (2017). Long noncoding RNA UCA1 induced by SP1 promotes cell proliferation via recruiting EZH2 and activating AKT pathway in gastric cancer. Cell Death Dis. 8 (6), e2839. 10.1038/cddis.2017.143 28569779 PMC5520878

